# Surface-Functionalized Hyperbranched Poly(Amido Acid) Magnetic Nanocarriers for Covalent Immobilization of a Bacterial γ*-*Glutamyltranspeptidase

**DOI:** 10.3390/molecules19044997

**Published:** 2014-04-22

**Authors:** Tzong-Yuan Juang, Shao-Ju Kan, Yi-Yu Chen, Yi-Lin Tsai, Min-Guan Lin, Long-Liu Lin

**Affiliations:** 1 Department of Applied Chemistry, National Chiayi University, Chiayi 60004, Taiwan; 2 Institute of Molecular Biology, Academia Sinica, Nankang District, Taipei 11529, Taiwan

**Keywords:** hyperbranched polymer, immobilization, magnetic nanocarrier

## Abstract

In this study, we synthesized water-soluble hyperbranched poly(amido acid)s (HBPAAs) featuring multiple terminal CO_2_H units and internal tertiary amino and amido moieties and then used them in conjunction with an *in situ* Fe^2+/^Fe^3+^ co-precipitation process to prepare organic/magnetic nanocarriers comprising uniformly small magnetic iron oxide nanoparticles (NP) incorporated within the globular HBPAAs. Transmission electron microscopy revealed that the HBPAA-γ-Fe_2_O_3_ NPs had dimensions of 6–11 nm, significantly smaller than those of the pristine γ-Fe_2_O_3_ (20–30 nm). Subsequently, we covalently immobilized a bacterial γ*-*glutamyltranspeptidase (*Bl*GGT) upon the HBPAA-γ-Fe_2_O_3_ nanocarriers through the formation of amide linkages in the presence of a coupling agent. Magnetization curves of the HBPAA-γ-Fe_2_O_3_/*Bl*GGT composites measured at 300 K suggested superparamagnetic characteristics, with a saturation magnetization of 52 emu g^−1^. The loading capacity of *Bl*GGT on the HBPAA-γ-Fe_2_O_3_ nanocarriers was 16 mg g^−1^ support; this sample provided a 48% recovery of the initial activity. The immobilized enzyme could be recycled 10 times with 32% retention of the initial activity; it had stability comparable with that of the free enzyme during a storage period of 63 days. The covalent immobilization and stability of the enzyme and the magnetization provided by the HBPAA-γ-Fe_2_O_3_ NPs suggests that this approach could be an economical means of depositing bioactive enzymes upon nanocarriers for *Bl*GGT-mediated bio-catalysis.

## 1. Introduction

In the quest to develop enzyme immobilization technologies, magnetic nanoparticles (NPs) are often used as nanocarriers because they allow facile re-collection and definite repeatability of biocatalysts having long-term stability [1,2,3,4,5,6,7]. The application of surface-functionalized iron oxide NPs for the covalent immobilization of enzymes is particularly attractive because it offers the possibility of linking soluble forms of enzymes covalently upon the nanomaterials. The challenge then becomes the identification of mild reaction conditions for anchoring large biomolecules onto NPs without disrupting their original conformations and biological functions [8]. Surface-functionalization of support materials has been investigated previously using dendritic polymers, including dendrimers and hyperbranched polymers (HBPs), that feature globular structures presenting multiple functional groups at their peripheries [9,10,11,12]. The architectures of dendritic polymers have attracted great attention for construction of three-dimensional (3D) systems having applications in drug delivery and as nanocarriers and organic/inorganic nanohybrids [13,14,15,16,17,18,19,20]. Surface-functionalized dendritic polymer/magnetic nanocarriers present many surfaces functional groups for covalent immobilization of natural enzymes, providing a means of facilitating product separation and sometimes improving the stability of biocatalysts. The ability of retain or recover the enzyme allows the biocatalyst to be separated from the product, thereby permitting continuous processes, and preventing carry-through of the protein or activity to subsequent process steps. A variety of modified nanomaterials, including graphene, gold NPs, layered silicates, and carbon nanotubes, have been also employed for the effective immobilization of enzymes [1,21,22,23,24,25,26].

Recently, we prepared water-soluble hyperbranched poly(amido acid)s (HBPAAs), based on wholly aliphatic structure with terminal carbonyl functionalities, through a facile self-condensation synthesis [27]. Considering their water-solubility and dendritic characteristics (size controllability; molecular uniformity), we wished to explore the possibility of using such HBPAAs to prepare organic/magnetic nanohybrids exhibiting desired magnetization for further biological application as enzyme nanocarriers. We suspected that employing these water-soluble HBPAAs as molecular templates would allow fine-tuning of the sizes of the resulting synthetic γ-Fe_2_O_3_ NPs. Indeed, we have used an *in situ* Fe^2+^/Fe^3+^ co-precipitation process to prepare organic/magnetic nanohybrids comprising uniformly small magnetic iron oxide NPs incorporated within globular HBPAAs for use as multifunctional nanocarriers. Transmission electron microscopy (TEM) revealed that our synthesized HBPAA-γ-Fe_2_O_3_ NPs had dimensions of 6–11 nm, significantly smaller than those of the pristine γ-Fe_2_O_3_ (20–30 nm). Next, we sought to immobilize a natural protein upon the multifunctional HBPAA-γ-Fe_2_O_3_ NPs as a route toward the development of magnetic biomaterial hybrids with potential applications in biocatalyst technology. Accordingly, we over-expressed and purified bacterial γ*-*glutamyltranspeptidase (*Bl*GGT) from the recombinant *E*. *coli* M15; this enzyme catalyzes the transfer of the γ-glutamyl moiety of glutathione to an amino acid, a short peptide, or water molecule [3]. We then covalently immobilized the enzyme *Bl*GGT upon the HBPAA-γ-Fe_2_O_3_ nanocarriers through the formation of amide linkages in the presence of *N*-ethyl-*N*-(3-dimethylaminopropyl)carbodiimide (EDC)/*N*-hydroxysuccinimide (NHS) as the coupling agent. Next, we studied the interactions of the magnetic NPs with the enzyme to realize the biocatalytic applications of these composites. Intramolecular forces between biomaterials and NPs may induce different catalytic functions for immobilized enzymes as a result of changes in their secondary structures, relative to those of their soluble free-enzyme counterparts. We have found, however, that covalent immobilization did not influence the stability of our tested enzyme; thus, the magnetization of the composites provides an economical means of preparing bioactive enzymes upon nanocarriers for *Bl*GGT-mediated bio-catalysis.

## 2. Results and Discussion

### 2.1. Preparation and Properties of the Synthesized Organic/Magnetic NPs

**Scheme 1:**
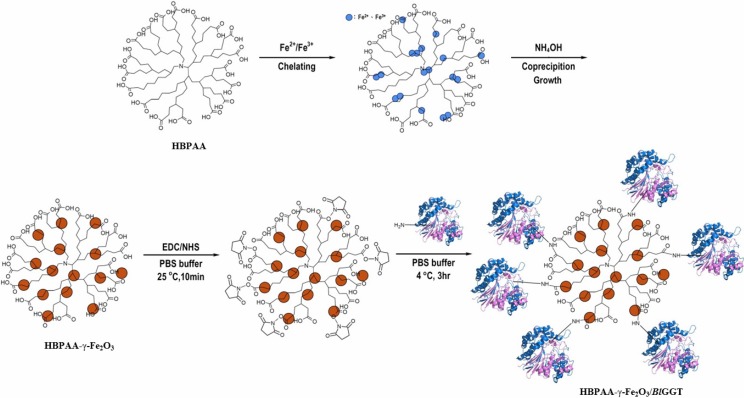
Preparation of HBPAA-γ-Fe_2_O_3_/*Bl*GGT-modified magnetic nanocarriers.

HBPs are highly branched 3D macromolecules that have attractive globular architectures. Previously, we used self-condensation of an AB_2_ monomer to prepare HBPAAs featuring wholly aliphatic backbones, multiple terminal CO_2_H units, and many internal tertiary amino and amido moieties [27]. In this study we encapsulated γ-Fe_2_O_3_ NPs through co-precipitation of Fe^2+^/Fe^3+^ in the presence of aqueous NH_4_OH and a templating water-soluble HBPAA having a molecular weight of 10,100 g mol^−1^ (Scheme 1). We then tested the γ-Fe_2_O_3_ NPs embedded within the water-soluble HBPAAs for use as magnetic nanocarriers. Accordingly, we covalently immobilized the enzyme *Bl*GGT upon the HBPAA-γ-Fe_2_O_3_ nanocomposite through the formation of amide linkages in the presence of EDC/NHS as a coupling agent.

**Figure 1:**
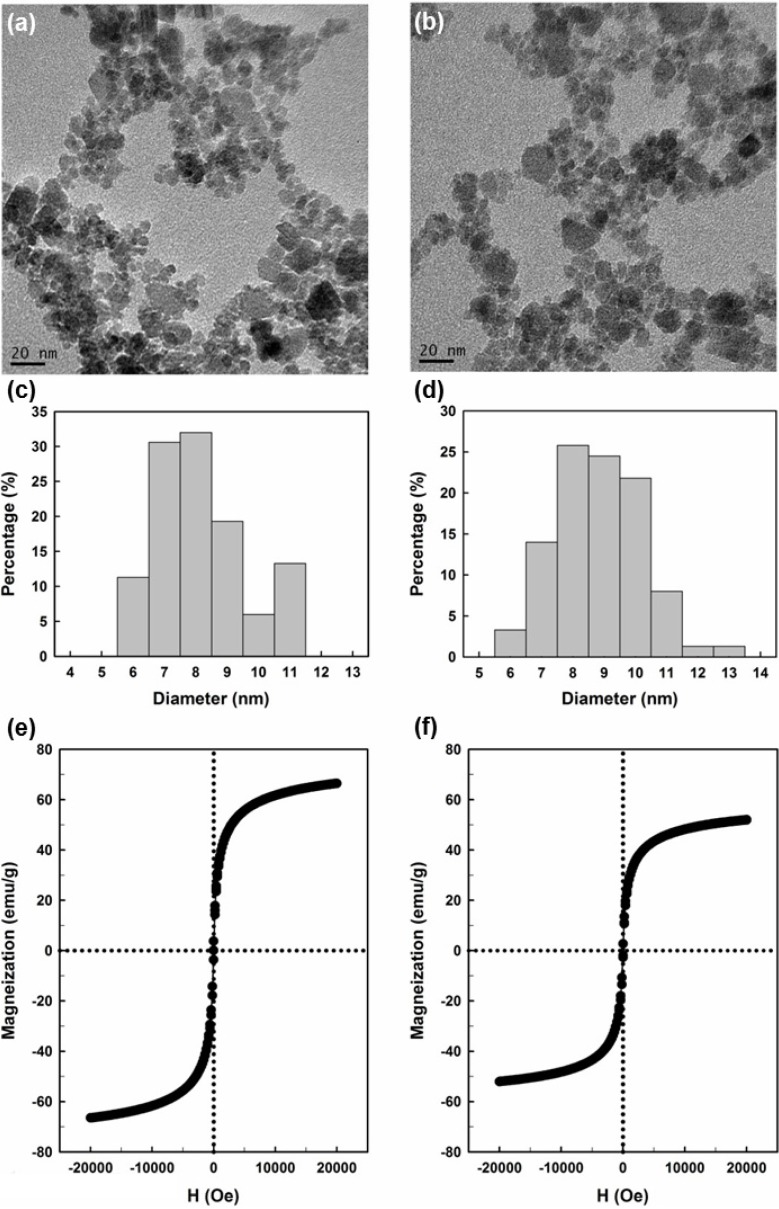
(**a**,**b**) TEM micrographs; (**c**,**d**) particle size distributions; and (**e**,**f**) magnetization curves of (**a**,**c**,**e**) HBPAA-γ-Fe_2_O_3_ and (**b**,**d**,**f**) HBPAA-γ-Fe_2_O_3_/*Bl*GGT.

Figure 1 displays TEM micrographs and magnetization curves for the free HBPAA-γ-Fe_2_O_3_ magnetic NPs and those covalently linked to *Bl*GGT. The HBPAA-γ-Fe_2_O_3_ magnetic NPs had a size distribution of 6–11 nm, significantly smaller than that (20–30 nm) of corresponding pristine γ-Fe_2_O_3_ prepared in the absence of HBPAA (Figure S1) [3,28]. The particle size distribution from a statistical sample of 150 particles in five TEM micrographs revealed a mean diameter of 8.3 ± 1.1 nm (Figure 1c). This globular HBPAA presents multiple COOH units, making it a useful template for the formation of modified magnetic NPs. The efficacy of the dendritic HBPAA templates in controlling the organic/magnetic nanohybrids was achieved through growth of the NPs within the branched structure, where they were protected by the exterior functional groups. The particle diameter is an important feature affecting the utility of an immobilization support. Indeed, smaller particles have larger surface-to-volume ratios and larger capacities to bind more units of enzymes on their surfaces, in addition to less restriction of the diffusion of the enzyme’s substrate and product. In our case, we found that the particles remained well dispersed after anchoring to *Bl*GGT; their mean diameter (9.3 ± 1.4 nm, Figure 1d) was slightly larger than that of the unlinked particles. Moreover, dynamic light scattering measurement also indicated the hydrodynamic size of HBPAA-γ-Fe_2_O_3_ and HBPAA-γ-Fe_2_O_3_/*Bl*GGT were 7.2 nm and 9.7 nm, respectively. (*i*.*e*., immobilization did not significantly change the average size of the magnetic NPs).

**Figure 2:**
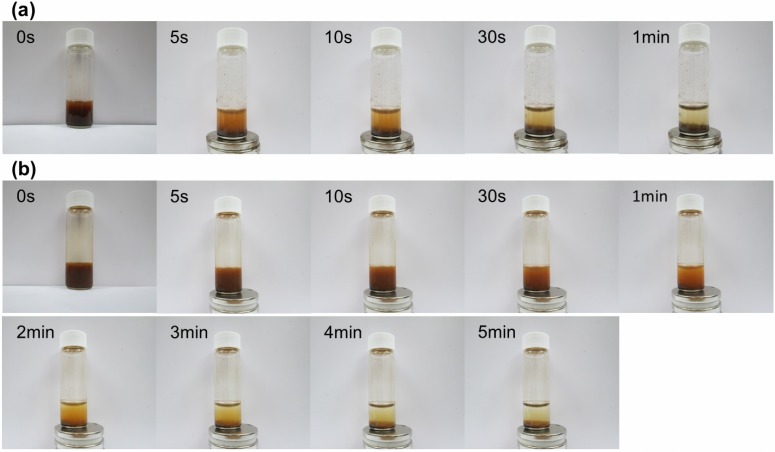
Magnetic behavior of (**a**) HBPAA-γ-Fe_2_O_3_ and (**b**) HBPAA-γ-Fe_2_O_3_/*Bl*GGT over time.

We used vibrating sample magnetometry to measure the magnetic moment of the surface-functionalized magnetic NPs under an applied field sweep from −20,000 to 20,000 Oe. The magnetization curves of HBPAA-γ-Fe_2_O_3_ and HBPAA-γ-Fe_2_O_3_/*Bl*GGT measured at 300 K revealed no detectable coercivity in the field sweep (Figure 1e, f), suggesting that the magnetic NPs were superparamagnetic materials; the saturation magnetizations of HBPAA-γ-Fe_2_O_3_ and HBPAA-γ-Fe_2_O_3_/*Bl*GGT were, however, different, with values of 67 and 52 emu g^−1^, respectively. The decrease in the magnetic moment of HBPAA-γ-Fe_2_O_3_/*Bl*GGT (22.4% of the value for HBPAA-γ-Fe_2_O_3_) was due to the attachment of units of the enzyme *Bl*GGT upon the magnetic NPs. The enzyme-anchored magnetic NPs formed brown suspensions in aqueous media; they were readily recovered from the solutions under the application of an external magnetic field, with a rapid response time (Figure 2). Once the external magnetic field was removed, slight shaking caused the functionalized NPs to redisperse into a suspension. The magnetization curves and the separation/redispersion process are consistent with the HBPAA-γ-Fe_2_O_3_/*Bl*GGT NPs having superparamagnetic properties.

**Figure 3:**
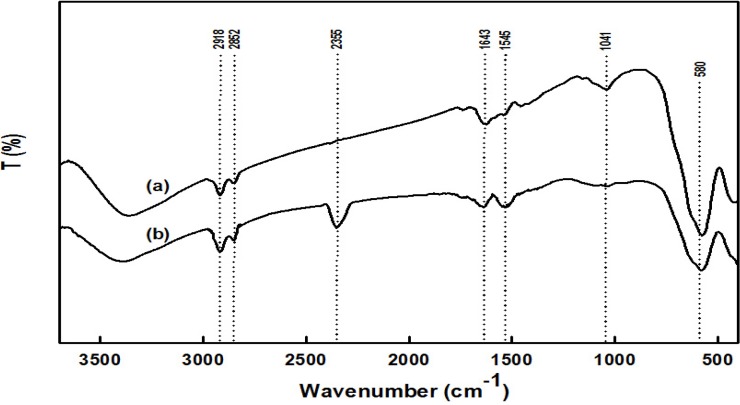
FTIR spectra of (**a**) HBPAA-γ-Fe_2_O_3_ and (**b**) HBPAA-γ-Fe_2_O_3_/*Bl*GGT.

FTIR spectroscopy confirmed the covalent linkage of *Bl*GGT to the HBPAA-functionalized magnetic NPs. Figure 3 displays the FTIR spectra of HBPAA-γ-Fe_2_O_3_ and HBPAA-γ-Fe_2_O_3_/*Bl*GGT. The peak at 580 cm^−1^ relates to the Fe–O group of γ-Fe_2_O_3_. The FTIR spectrum of HBPAA-γ-Fe_2_O_3_ (Figure 3a) features a strong signal for C=O stretching near 1643 cm^−1^ and a weak signal for NH bending near 1545 cm^−1^, typical of the secondary amide groups of HBPAA, suggesting the successful formation of HBPAA-encapsulated γ-Fe_2_O_3_ nanohybrids [27]. A strong characteristic band, which we assign to the NH and NH_2_ bending vibrations of the enzyme, appears at 1545 cm^−1^ in the spectrum of HBPAA-γ-Fe_2_O_3_/*Bl*GGT (Figure 3b). Moreover, an absorption near 2355 cm^−1^ emerged, attributable to coupling of an asymmetric NH stretching vibration and a CH stretching vibration [29]. The signals at 1545 and 2355 cm^−1^ had stronger intensities when we anchored more of the enzyme *Bl*GGT onto the surface of HBPAA-γ-Fe_2_O_3_ as the reaction ratio of *Bl*GGT/HBPAA-γ-Fe_2_O_3_ (*w/w*) of 0.5. These spectroscopic data are consistent with the *Bl*GGT molecules having been successfully appended to the HBPAA-functionalized magnetic NPs.

**Figure 4:**
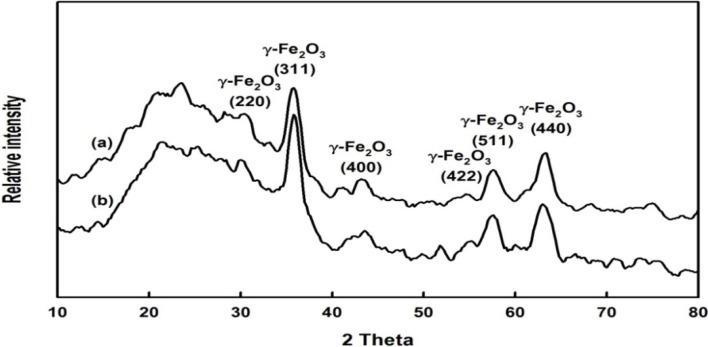
XRD patterns of (**a**) HBPAA-γ-Fe_2_O_3_ and (**b**) HBPAA-γ-Fe_2_O_3_/*Bl*GGT.

Figure 4 displays powder XRD patterns of HBPAA-γ-Fe_2_O_3_ and HBPAA-γ-Fe_2_O_3_/*Bl*GGT. The five peaks that appear at values of 2*θ* of 30, 35, 43, 57, and 63° correspond to the (2 2 0), (3 1 1), (4 0 0), (5 1 1), and (4 4 0) planes, respectively, of γ-Fe_2_O_3_ [2].

All of the peaks perfectly matched with the inverse spinel structure of pristine γ-Fe_2_O_3_. This observation confirms that surface modification of magnetite with HBPAA did not lead to any impure crystal phase corresponding to hematite NPs. We calculated the average crystallite size using Scherrer equation (*D* = *kλ*/*β*cos*θ*, where *k* is the shape factor, *λ* is the wavelength of the X-rays, *β* is the full width at half maximum of the peak, and *θ* is the Bragg angle). The average crystallite sizes of HBPAA-γ-Fe_2_O_3_ and HBPAA-γ-Fe_2_O_3_/*Bl*GGT—5.25–6.62 and 5.61–8.09 nm (Table S1), respectively—indicated that the HBPAA-modified NPs were uniform. The cartoon representation in Scheme 1 illustrates the dendritic HBPAA functioning as templates for the co-precipitation of the Fe^2+^/Fe^3+^ ions, generating the new type of organic/magnetic nanohybrids. We suspect that the tertiary amino groups chemically bonded with the ferric ions, thereby affecting the subsequent nucleation and growth of the γ-Fe_2_O_3_ NPs, although determining the exact mechanism will require further analysis. The multiple exterior polar terminal CO_2_H units extended into the aqueous phase, sterically stabilizing the magnetic NPs from collision and flocculation. After co-precipitation, the magnetic NPs were present as a homogeneous distribution within the HBPAA structures. The diameter of each HBPAA-γ-Fe_2_O_3_ and HBPAA-γ-Fe_2_O_3_/*Bl*GGT particle was less than 10 nm.

### 2.2. Immobilization of the Recombinant Enzyme

**Figure 5:**
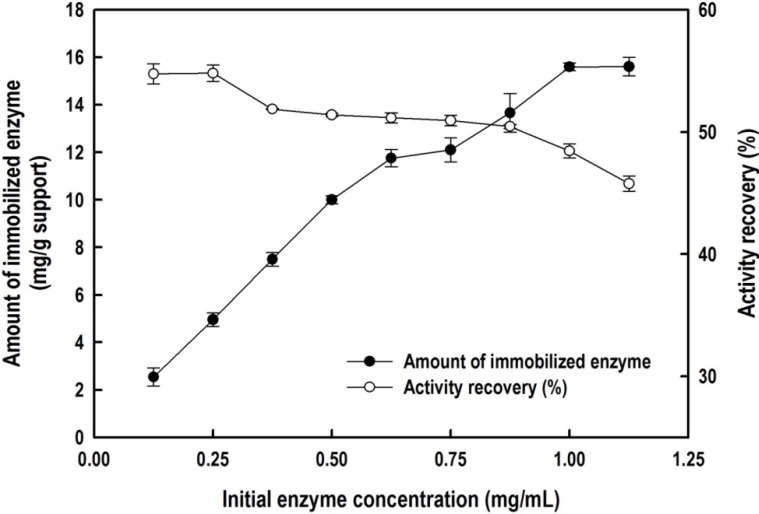
Effect of initial *Bl*GGT concentration on the amount of immobilized enzyme (**closed circles**) and recovery of the activity (**open circles**) of HBPAA-γ-Fe_2_O_3_/*Bl*GGT.

To ensure that the immobilized biocatalyst had a high enzyme loading and a high recovery of activity, we investigated the effects of the initial enzyme concentration and coupling time on the immobilization efficiency. Figure 5 displays the effect of the initial *Bl*GGT concentration on the amount of the biocatalyst covalently attached to the HBPAA-encapsulated magnetic NPs and on the recovery of the enzymatic activity. The amount of immobilized enzyme increased almost proportionally to the initial enzyme concentration in the bulk solution from 0.125 to 1.00 mg mL^−^^1^, but leveled off thereafter. Eventually, the maximum loading of *Bl*GGT reached 16.2 mg g^−^^1^ of support, where it provided a recovery of activity of 47.4%. The recovery of the activity of *Bl*GGT, defined as the ratio of the GGT activity of the immobilized enzyme to the initial activity, remained almost unchanged for low initial concentrations of the enzyme (0.125–0.250 mg mL^−^^1^); further increases in the initial enzyme concentration, however, decreased the recovery of activity. A possible explanation for this behavior is that multiple layers of enzyme molecules formed on the surface of the magnetic NPs at high enzyme loadings, thereby blocking the active sites and limiting the diffusion of the substrate.

**Figure 6:**
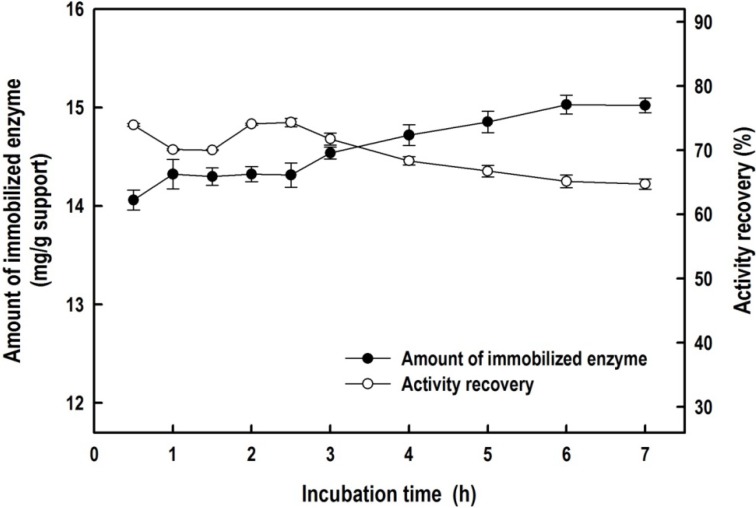
Effect of coupling time on the amount of immobilized BlGGT (**closed circles**) and the recovery of the activity (**open circles**) for HBPAA-γ-Fe_2_O_3_/BlGGT; initial enzyme concentration: 0.8 mg mL^−1^.

Figure 6 presents the effect of the coupling time on the immobilization efficiency of *Bl*GGT on the HBPAA-functionalized magnetic NPs. The amount of immobilized enzyme increased upon increasing the coupling time, with the maximum amount of immobilized enzyme being 15.1 mg g^−^^1^ of support. The recovery of the activity of the immobilized *Bl*GGT remained almost constant (*ca.* 74.7%) for incubation times up to 2.5 h, gradually decreasing thereafter. Thus, long immobilization times decreased the recovery of the activity, presumably because of the creation of too many covalent linkages between the HBPAA-γ-Fe_2_O_3_ NPs and the *Bl*GGT enzyme, thereby disturbing the conformation of the biocatalyst.

### 2.3. Characterization of Free and Immobilized Enzymes

**Figure 7:**
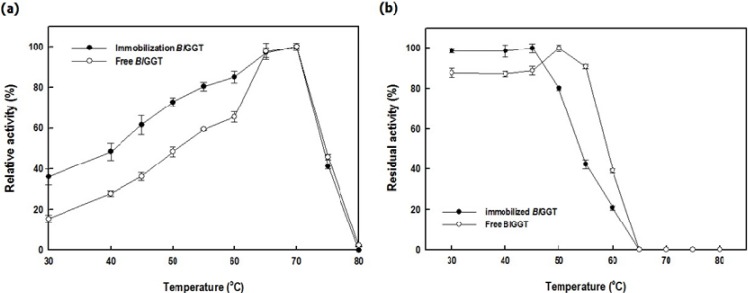
Effect of temperature on the (**a**) activity and (**b**) stability of the free and immobilized enzyme. Data are means from three independent experiments; standard deviations were less than ±4.7%.

Figure 7a reveals that the optimal temperature for enzymatic reactions for the free *Bl*GGT and immobilized enzyme was 70 °C. We also measured the thermal stability of the free and immobilized *Bl*GGTs after incubating the enzyme samples at temperatures in the range 30–80 °C and then measuring their residual activity. In Figure 7b, both the free and immobilized enzymes display similar profiles, with the former exhibiting slightly higher thermal stability. Thermal stabilization through immobilization has been observed for a number of enzymes conjugated to magnetic NPs [4]. The reasons for the absence of any significant thermal stabilization of *Bl*GGT after immobilization remain to be elucidated.

**Figure 8:**
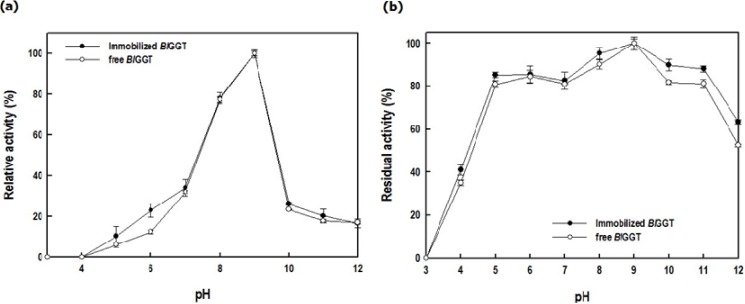
Effect of pH on the (**a**) activity and (**b**) stability of the free and immobilized enzymes. Data are means from three independent experiments; standard deviations were less than ±6.1%.

Figure 8 reveals the effect of pH on the enzymatic activity of the immobilized *Bl*GGT. The optimal pH of immobilized *Bl*GGT was 9.0, almost the same as that of the free enzyme (Figure 8a). The stability of the immobilized *Bl*GGT was slightly higher than that of free enzyme in the pH range from 6 to 11 (Figure 8b). Practically, it would be ideal if the immobilized enzyme would function over a wide range of values of pH.

**Figure 9:**
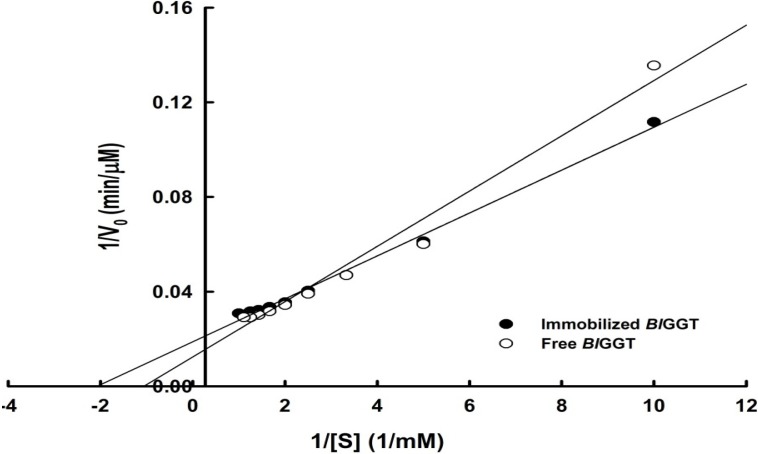
Lineweaver–Burk plots for the free and immobilized enzymes.

We employed Lineweaver–Burk plots to obtain the kinetic parameters for the transpeptidation reactions of l-γ-Glu-*p*-NA free and immobilized enzymes (Figure 9). The Michaelis–Menten constants, *K*_M_ and *V*_max_, for the free enzyme were 0.94 mM and 80.64 µmol min^−1^ mg^−1^, respectively (Table 1). The immobilized *Bl*GGT had an apparent value of *K*_M_ of 0.48 mM and a value of *V*_max_ of 53.19 µmol min^−1^ mg^−1^, thereby decreasing the catalytic efficiency (*k*_cat_/*K*_m_) from 33.70 mM^−1^ s^−1^ for the free enzyme to 14.85 mM^−1^ s^−1^ for the immobilized enzyme. Thus, the immobilization process decreased the substrate affinity of *Bl*GGT significantly and may also have hindered the accessibility of the reactants to the active site. These results are consistent with the kinetic observations of other enzymes immobilized on magnetic nanomaterials [4,5,28].

**Table 1:** Kinetic properties of the free and immobilized enzymes.

Enzyme	Specific activity (U mg^−1^)	*V*_max_ (µM min^−1^)	*K*_m_ (mM)	*k*_cat_ (s^−1^)	*k*_cat_/*K*_m_ (mM^−1^ s^−1^)
Immobilized *Bl*GGT	4.24 ± 0.08	53.19	0.48	7.13	14.85
Free *Bl*GGT	12.08 ± 0.09	80.64	0.94	31.68	33.70

### 2.4. Reusability

**Figure 10:**
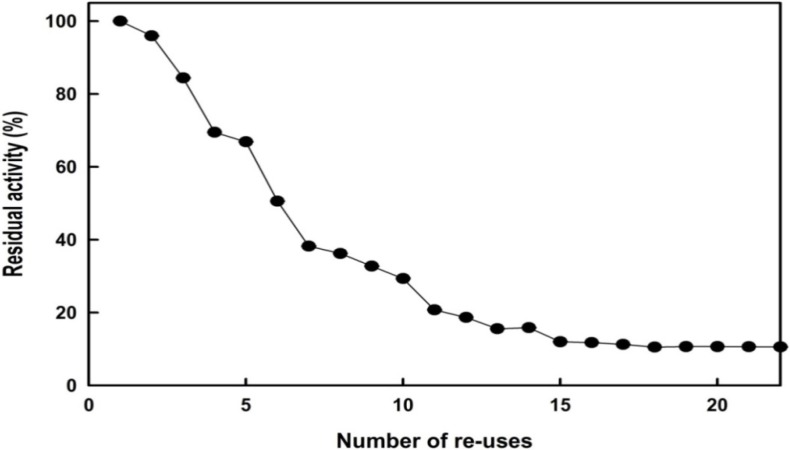
Operational stability of immobilized enzymes.

The duration of a biocatalyst is an important factor affecting its potential industrial applications. Accordingly, we evaluated the operational stability of the immobilized *Bl*GGT in a repeated batch process. After each cycle, we recovered the immobilized enzyme through magnetic separation and recycled it for the transpeptidation reaction of l-γ-Glu-*p*-NA. Figure 10 reveals that the GGT activity of the immobilized enzyme did not decrease significantly after the first cycle; indeed, it retained approximately 32% of its initial activity after 10 cycles. Given that *Bl*GGT was stable for 24 h at 40 °C (data not shown), we attribute the gradual loss of GGT activity to conformational changes of the immobilized enzyme upon reuse or to a distributive stability of each enzyme, due to differences in the number of covalent bonds between the enzyme units and the support matrix.

### 2.5. Storage Stability of Free and Immobilized Enzymes

**Figure 11:**
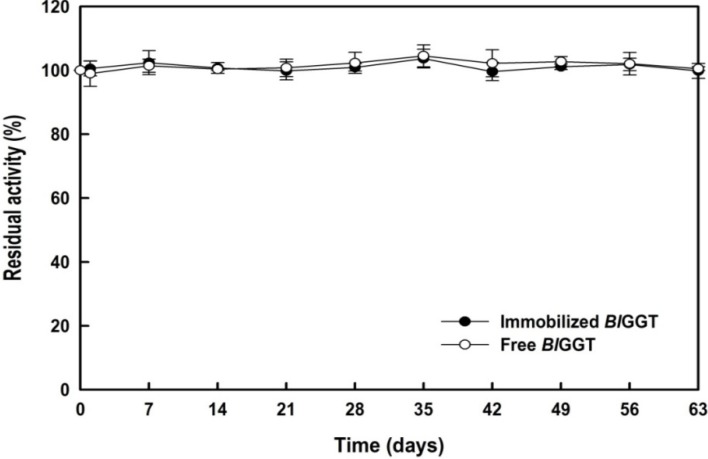
Storage stability of free and immobilized enzymes. Data are means from three independent experiments; standard deviations were less than ±4.2%.

The storage stability of an enzyme is important criterion for biocatalyst-mediated processes because it affects the economics of industrial bioprocesses, which are closely tied to the production cost of the enzyme. Therefore, we evaluated the storage stability of the free and immobilized enzymes (Figure 11). After an incubation period of 63 days, the residual activities of the free and immobilized enzymes were 98.2% and 98.3%, respectively. These experimental findings suggest that *Bl*GGT remained quite stable after its anchoring to the surface-modified magnetic HBPAA-γ-Fe_2_O_3_ NPs. Thus, the covalent-immobilization of *Bl*GGT onto the HBPAA-γ-Fe_2_O_3 _NPs was successful at incorporating the active natural protein in the nanostructures, suggesting that such HBPAA-encapsulated iron oxide structures are promising nanocarriers.

## 3. Experimental

### 3.1. Materials

EDC, NHS, ferric chloride hexahydrate, and ferrous chloride tetrahydrate were obtained from Aldrich (St. Louis, MO, USA). The chemical compounds required for the enzyme assay, including l-γ-glutamyl-*p*-nitroanilide Is same company, combine lists(l-γ-Glu-*p*-NA), glycylglycine (Gly-Gly), and *p*-nitroaniline (*p*-NA), were purchased from Sigma-Aldrich Fine Chemicals (St. Louis, MO, USA). All other chemicals were commercial products of analytical or molecular biological grade. HBPAA (molecular weight: 10,100 g mol^−1^) was synthesized according to a procedure described previously [27].

### 3.2. HBPAA-γ-Fe_2_O_3_ Magnetic Nanohybrids

For the preparation of HBPAA-γ-Fe_2_O_3_ nanohybrids at a 1:1 HBPAA–to–iron oxide weight ratio, a homogeneous dispersion of HBPAA [0.1 g in D.I. water (5 mL)] was adjusted to pH 2. A mixture of 0.43 M Fe^2^^+^ (1 mL) and 0.86 M Fe^3^^+^ (1 mL) was added to the dispersion (*i*.*e*., at a 1:2 molar ratio of Fe^2^^+^/Fe^3^^+^) and stirred vigorously for 1 h. The addition was followed by a portion of aqueous NH_4_OH (0.70 N) until the pH of the dispersion reached approximately 9–10, and then heat at 80 °C for 3 h. The precipitate was separated through three cycles of centrifugation and washing with deionized water and then it was air-dried at room temperature. Using a similar procedure, pristine γ-Fe_2_O_3_ magnetic NPs were prepared without the stabilizing HBPAAs.

### 3.3. HBPAA-γ-Fe_2_O_3_/BlGGT-Modified Magnetic Nanocarriers

To prepare enzyme-anchored HBPAA-γ-Fe_2_O_3_ magnetic NPs, purified *Bl*GGT was immobilized on the HBPAA-γ-Fe_2_O_3_ magnetic NPs with the *Bl*GGT/HBPAA-γ-Fe_2_O_3_ (*w/w*) ratio of 0.5 through the formation of amide linkages in the presence of NHS/EDC as the coupling agent. The mixture was allowed to stir at room temperature for 24 h. The enzyme immobilized HBPAA-γ-Fe_2_O_3_ NPs were allowed to separate by centrifuges for 10 min at 10,000 rpm and the soluble portion was preserved for the estimation of enzyme concentration. The binding percentage of *Bl*GGT was estimated by determining the amount of protein in the unreacted fraction. The protein concentration was determined through the colorimetric method (measurement at 595 nm) using a Bio-Rad protein assay kit and bovine serum albumin as the reference standard.

### 3.4. Enzyme Activity

The γ*-*glutamyltranspeptidase (GGT) enzyme activity was assayed at 40 °C according to the method of Orlowski and Meister [30]; the formation of *p*-NA was recorded by monitoring the absorbance change at 410 nm. The reaction mixture contained 1.25 mM l-γ-Glu-*p*-NA, 30 mM Gly-Gly, 1 mM MgCl_2_, 20 mM Tris-HCl buffer (pH 8.0), the enzyme in solution (20 μL) at a suitable dilution, and enough distilled water to bring the final volume to 1 mL. One unit of GGT activity is defined as the amount of enzyme required to produce 1 μmol of *p*-NA per minute under the assay conditions. The kinetic parameters for the free and immobilized enzymes were estimated by measuring the production of *p*-NA in 20 mM Tris-HCl buffer (pH 8.0, 1 mL) containing various concentrations of l-γ-Glu-*p*-NA (0.1–2.0 *K*_M_), 30 mM Gly-Gly, 1 mM MgCl_2_, and appropriate amounts of the free and immobilized enzymes. The values of *K*_M_ and *V*_max_ were calculated from the slope and *y*-axis intercept, respectively, of the Lineweaver–Burk plots.

### 3.5. Effects of Temperature and pH

The effect of temperature on the free and immobilized enzymes was evaluated by incubating free *Bl*GGT (8.7 U mL^−1^) and *Bl*GGT-conjugated HBPAA-γ-Fe_2_O_3_ magnetic NPs (34.1 mg; wet weight) in 20 mM Tris-HCl buffer (pH 8.0, 10 mL) at various temperatures (30–80 °C) for 10 min. The GGT activity was determined according to the assay procedure described above. The thermal stabilities of the free and immobilized enzymes were measured in 20 mM Tris-HCl buffer (pH 8.0, 10 mL) at various temperatures in the range 30–80 °C. After 30 min of incubation, the residual activity was determined under the standard assay conditions. The experiments were performed in triplicate; data are expressed as mean values.

To investigate the effect of pH on the free and immobilized enzymes, *Bl*GGT (8.7 U mL^−1^) and *Bl*GGT-conjugated HBPAA-γ-Fe_2_O_3_ magnetic NPs (34.1 mg; wet weight) were incubated at 40 °C with 10 mL of 20 mM Tris-maleate buffer (pH 4.5–6.0), 20 mM potassium phosphate buffer (pH 6.0–8.0), 20 mM Tris-HCl buffer (pH 8.0–9.0), or 20 mM glycine-NaOH buffer (pH 9.0–11.0); the GGT activity was determined according to the standard assay conditions. For measurements of pH-stability, the free and immobilized enzymes were maintained at 4 °C for 30 min in the different buffers. The residual GGT activity was determined under the standard assay conditions. The experiments were performed in triplicate; data are expressed as mean values.

### 3.6. Reusability of Immobilized Enzyme

The immobilized *Bl*GGT was repeatedly used to catalyze the transpeptidation of l-γ-Glu-*p*-NA in a batch process. Enzyme-conjugated HBPAA-γ-Fe_2_O_3_ magnetic NPs (12 mg) in 20 mM Tris-HCl buffer (pH 8.0, 1 mL) containing 1.25 mM l-γ-Glu-*p*-NA, 30 mM Gly-Gly, 1 mM MgCl_2_, and 20 mM Tris-HCl buffer (pH 8.0) were shaken (100 rpm) at 40 °C for 10 min each time. The GGT activity was immediately determined under the standard assay conditions. After each cycle, the enzyme/matrix complex was washed twice with 20 mM Tris-HCl buffer (pH 8.0, 1 mL) and reused for the next run. The experiments were performed in triplicate; data are expressed as mean values.

### 3.7. Storage Stability of the Immobilized Enzyme

The operational stability was assessed for the immobilized *Bl*GGT (*ca.* 1.2 g, wet weight) during storage in 20 mM Tris-HCl (pH 8.0, 100 mL) at 4 °C. At specific time intervals, aliquots (1 mL) were withdrawn to determine the GGT activity under the standard assay conditions. The experiments were performed in triplicate; data are expressed as mean values.

### 3.8. Measurements

The covalent immobilization of *Bl*GGT on the HBPAA-functionalized magnetic NPs was confirmed using FTIR spectroscopy (KBr disc method) in the range 4000–500 cm^−1^. FTIR spectra were recorded using a Jasco 4100 FTIR Spectrophotometer (Jasco, Easton, MD, USA) equipped with a Jasco ATR Pro 450-S accessory. Powder X-ray diffractometry (XRD; model D-MAX-2B, Rigaku, TX, USA) was performed in the 2*θ* range from 10 to 80° at a scan rate of 3°/min. The crystallite size of the magnetite was determined by applying the Scherrer formula for each peak; the mean value was then calculated. The average hydrodynamic particle size was estimated using a particle size analyzer (90 Plus Brookhaven Instrument Corp., Holtsville, NY, USA) equipped with a 15 mW solid-state laser (675 nm). The saturation magnetization of the synthesized nanohybrids was measured using a superconducting quantum interference device magnetometer (SQUID, MPMS7) at 300 K under an applied magnetic field of ±20,000 Oe. TEM images of the samples transferred to carbon-coated Cu grids were recorded using a Zeiss EM 902A microscope (JEOL Co. Ltd, Tokyo, Japan) operated at an accelerating voltage of 80 kV.

## 4. Conclusions

We have examined the efficacy of water-soluble HBPAA molecules as dendritic templates for the preparation of organic/magnetic nanohybrids through the growth of γ-Fe_2_O_3_ NPs within their branched structures, with steric protection provided by the exterior functional groups. The globular HBPAA acted as a molecular container that allowed tuning of the intermolecular interactions at the interface of the growing γ-Fe_2_O_3_ NPs, thereby decreasing the particle size to 6–11 nm relative to that (20–30 nm) of the pristine γ-Fe_2_O_3_. The multiple functional groups presented by the resulting HBPAA-γ-Fe_2_O_3_ NPs allowed efficient covalent immobilization of the enzyme *Bl*GGT, providing a loading capacity of 16 mg g^−1^ support and an initial activity of 48%. Moreover, the presence of the magnetic NPs allowed ready recovery and reuse of the grafted catalyst for at least 10 cycles with 32% of the initial activity. This report is the describing the covalent immobilization of a bioactive enzyme upon dendritic macromolecule–modified magnetic NPs. The immobilized enzyme remained thermally stable and storage-stable, suggesting that this approach could make expensive enzymes become economically viable, thereby opening up new biotechnological horizons for GGT-mediated catalysis.

## Supplementary Materials

Supplementary materials can be accessed at: http://www.mdpi.com/1420-3049/19/4/4997/s1.
