# Supplementary material for: Surface-Functionalized Hyperbranched Poly(Amido Acid) Magnetic Nanocarriers for Covalent Immobilization of a Bacterial γ-Glutamyltranspeptidase
Source: Molecules. 2014 Apr 22;19(4):4997–5012. doi: 10.3390/molecules19044997 (PMC6271278; doi:10.3390/molecules19044997)

Supplementary Materials

Table S1. Grain size parameters calculated from peak width data and thetas for HBPAA-γ-Fe_2O_3 and HBPAA-γ-Fe_2O_3/*Bl*GGT.

Sample	*β*	2*θ*	*D (nm)*[a]
	HBPAA-γ-Fe_2O_3		
1	0.022	35.8	6.62
2	0.031	63.2	5.25
	HBPAA-γ-Fe_2O_3/*Bl*GGT		
3	0.018	35.8	8.09
4	0.029	63.0	5.61

[a] Calculated from Scherrer equation: $D = k\lambda/\beta\cos\theta$, where k is the shape factor, λ is the wavelength of the X-rays, β is the full width at half maximum of the peak, and θ is the Bragg angle.

Figure S1. TEM micrographs of iron oxide NPs: (**a**) pristine iron oxide, revealing clusters of particle aggregates (particle size: 20–30 nm) and (**b**) HBPAA-Fe_3O_4 (1:1, *w/w*), revealing iron oxide particles (particle size: 6–11 nm).

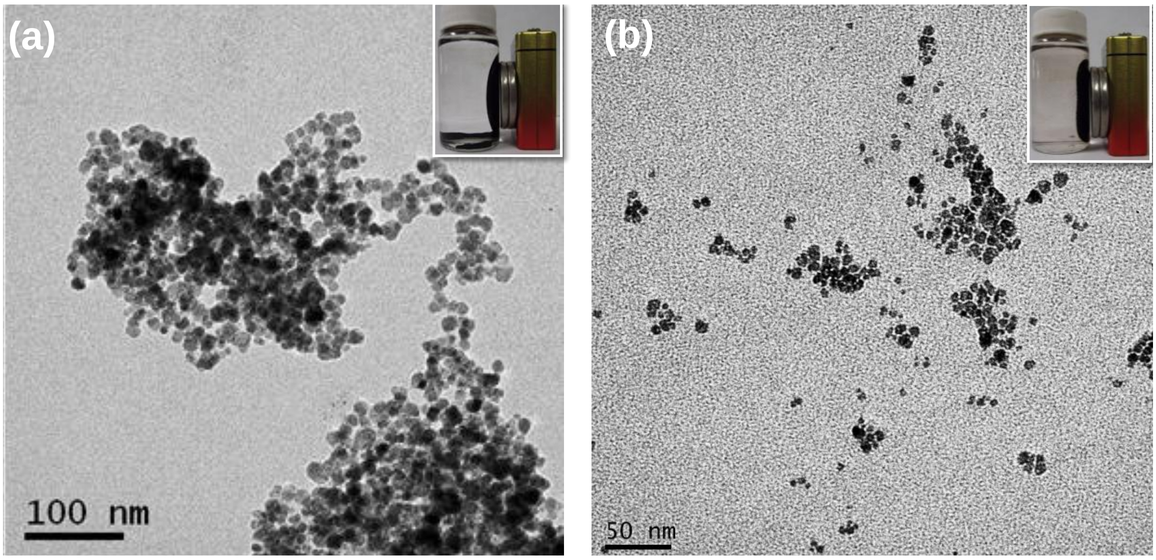

Supplement: Supplementary file 1 [file molecules-19-04997-s001.pdf]
